# Inside Doctor Livingstone: a Scottish icon's encounter with tropical disease

**DOI:** 10.1017/S003118201600202X

**Published:** 2016-12-08

**Authors:** MICHAEL P. BARRETT, FEDERICA GIORDANI

**Affiliations:** Wellcome Trust Centre for Molecular Parasitology, Institute of Infection, Immunity and Inflammation, College of Medical, Veterinary and Life Sciences, University of Glasgow, Glasgow G12 8TA, UK

**Keywords:** Dr Livingstone, Parasitology, Tropical medicine, African exploration, tsetse fly, tampan tick, helminthology, schistosomiasis, malaria, quinine

## Abstract

Dr David Livingstone died on May 1st 1873. He was 60 years old and had spent much of the previous 30 years walking across large stretches of Southern Africa, exploring the terrain he hoped could provide new environments in which Europeans and Africans could cohabit on equal terms and bring prosperity to a part of the world he saw ravaged by the slave trade. Just days before he died, he wrote in his journal about the permanent stream of blood that he was emitting related to haemorrhoids and the acute intestinal pain that had left him incapable of walking. What actually killed Livingstone is unknown, yet the years spent exploring sub-Saharan Africa undoubtedly exposed him to a gamut of parasitic and other infectious diseases. Some of these we can be certain of. He wrote prolifically and described his encounters with malaria, relapsing fevers, parasitic helminths and more. His graphic writing allows us to explore his own encounters with tropical diseases and how European visitors to Africa considered them at this time. This paper outlines Livingstone's life and his contributions to understanding parasitic diseases.

## A CELEBRATED ICON

Livingstone has been the subject of a multitude of biographies, including (Jeal, 1973; Ross, 2002) and many aspects of his work and life are now collected into a major online resource (www.livingstoneonline.org). With the bicentenary of his birth in 2013 a number of new materials and scholarship were produced, including a special issue of the Scottish Geographical Journal, whose contents were summarized along with a note that the complexities and unique position of Livingstone in history are creating an expanding field of ‘Livingstone studies’ (Livingstone, 2013). An exhibition celebrating his life was held at the National Museum of Scotland and a book of essays updated on many aspects of Livingstone's contributions (Worden, 2013), including an assessment of his contributions to the medical sciences (Harrison, 2013). Here we will give only a brief overview of the key biographical facts before providing specific insight into his encounters with parasitic disease.

## EARLY LIFE AND MEDICAL TRAINING

**Fig. 1.**
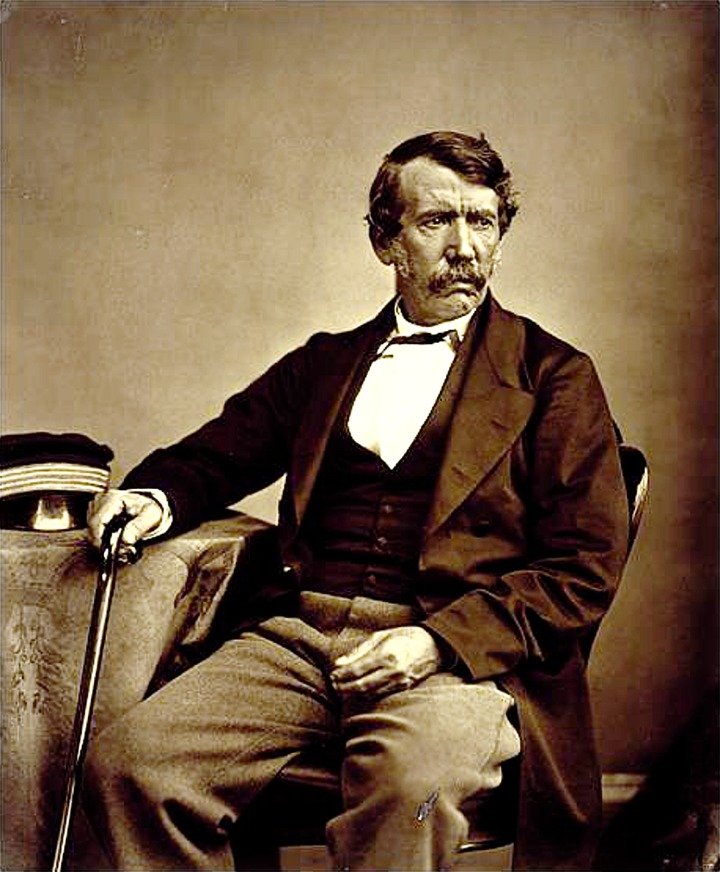
David Livingstone, photographed by Thomas Annan in 1864. Used by kind permission of Flickr The Commons.

Born in 1813 to Neil and Agnes Livingstone at the cotton works of Henry Montieth & Co. in Blantyre, near Glasgow, David Livingstone (Fig. 1) was one of five children who survived to adulthood. Although Montieth's mill had unusually enlightened ways for the time, Livingstone's upbringing was impoverished. He started work in the mill from the age of 10 as a piecer, picking up small bits of cotton from the mill floor to recycle. Work began at six in the morning and continued till eight in the evening. However, he could attend school for few hours after work and then read late into the night, teaching himself Latin and Greek, for example. He became fascinated by natural history, but only when he persuaded his father that learning medicine would allow him to perform God's work among the heathen, did he eventually enrol at Anderson's University in Glasgow (the predecessor of Strathclyde University) in 1836. Here, Livingstone made crucial friends, not least James ‘Paraffin’ Young, who made a fortune from the production of shale-oil across Scotland. Interestingly, the Andersonian College, as the institution was also called, moved to new premises in 1889 and it was here that the Wellcome Trust Centre for Molecular Parasitology (WTCMP) was housed in the 1990s. Over his 2 years at medical school Livingstone completed courses in Chemistry, Anatomy, Surgery and Materia Medica. He also attended lectures in Greek at the University of Glasgow, while studying Theology at the Congregational Church. The University of Glasgow awarded him an LLD (Doctorate of Laws) in 1854 and he is counted among the University's most revered alumni.

## MISSIONARY TRAVELS

In 1838, Livingstone joined the London Missionary Society (LMS), honouring his promise to learn medicine to put to use in missionary work. Based at Chipping Ongar, in Essex, his theological teachings were complemented by continued medical studies in London, including at Charing Cross and Moorfields hospitals. Unable to afford to sit the exams in London, he returned to Glasgow, where he was awarded a license to practice medicine by the Faculty of Physicians and Surgeons of Glasgow (now the Royal College of Physicians and Surgeons) in 1840. Although he had initially planned to work as a missionary in China, the outbreak of the Opium Wars precluded this. Instead, after meeting Dr Robert Moffat, a missionary based in Kuruman, South Africa, Livingstone was persuaded that Africa would offer great opportunities for his work. Once ordained by the LMS as a missionary in 1841, he left for the Cape. As an employee of the LMS, his first trip to Africa was ostensibly to convert Africans to Christianity. However, as a preacher he was poor and he failed to make a single true convert (Sechele, chief of the Bakwena, claimed a conversion, but breaking with polygamy proved too difficult for him to be considered a true Christian). Notwithstanding, Sechele did promote Christianity and evangelising missionaries that followed Livingstone were hugely influential in establishing this religion, which continues as the dominant one across Southern Africa. Having moved north of Moffat's mission station at Kuruman in South Africa, in 1844 a lion attack left Livingstone with a broken arm. While being nursed back to health by Mary, Moffat's daughter, he proposed and they married. They set up new mission stations and started family life. However, when their baby daughter Elizabeth died in 1850, Mary and their other four children returned to Britain. By then, Livingstone had already crossed the Kalahari Desert to Lake Ngami (in 1849) and he was now determined to find waterways to bring Europeans into Central Africa. With this in mind, he walked first to Luanda on the West Coast (in modern Angola), and then, believing this route was too treacherous to offer a suitable means to enter, he set off on his East to West trek, following the course of the Zambesi river, famously passing Mosi-oa-Tunya (‘The Smoke that Thunders’), which he named Victoria Falls. The Zambesi, he believed, offered the ideal route to bring Europeans into Africa whereby Christianity, Commerce and Civilization would lead to an end to the slave trade he had seen decimating the Continent. In 1856, he returned to Britain, where he quickly wrote ‘Missionary Travels and Researches in South Africa’ (Livingstone, 1857). The book became a sensation, sparking enormous interest in the exotic world of Africa and propelling Livingstone to fame.

## THE ZAMBESI EXPEDITION

The British public, increasingly interested in Empire, new lands and far-away places, offered hero status to the Missionary. In 1858, he set off on a second, scientific expedition, funded by the Government, to formally prospect for the economic potential of Central Africa and explore more about how the Zambesi might function as a gateway to the region. The expedition was widely considered an abject failure, seemingly having failed in what the British public had considered its primary motivation: opening Africa to British colonization. Indeed, the expedition was fraught with difficulty. Livingstone could not get on with his colleagues and one by one they left. Many missionaries, naively following in his footsteps, arrived only to succumb to malaria, a fate shared, on April 27th, 1862, by Mary Livingstone, who had returned to Africa, desperate to reunite with her husband. The death of his wife affected the Doctor profoundly, in spite of the neglect he had shown her (Healey, 1988; Forster, 2001; Davidson, 2012). Malaria was a major cause for the expedition's failure, its members being frequently prostrated and unable to work. Extraordinarily, Livingstone considered idleness and indiscipline to be major contributing factors, accusing his companions of laziness. He actually dismissed two young members of the expedition, Thomas Baines and Richard Thornton, on those grounds. John Kirk, the expedition doctor and economic botanist, was the only one of the party who could tolerate Livingstone's extreme behaviour to any extent, and yet he too privately despaired of his leader,

‘*The infatuation which blinds him, I cannot comprehend*,’

Kirk wrote in his journal,

‘*It seems madness and to follow a man running such risks for the empty glory of geographical discovery is more than I would consent to … I can come to no other conclusion than that Dr L is out of his mind*’.

Lawrence Dritsas has recently offered a more objective account of the expedition's scientific outcomes (Dritsas, 2010). With hindsight, the discoveries and their reporting were extraordinary. Nearly 100 scientific papers were published, describing the geology, anthropology, flora and fauna of Southern Africa. Returning to Britain in 1864, at the insistence of the Government, however, Livingstone was received with none of the fanfare of his previous homecoming. With his brother Charles, he nevertheless wrote another best-selling book summarizing the expedition (Livingstone and Livingstone, 1865).

## LAST JOURNEYS

In spite of the perceived failure of the Zambesi expedition, by the 1860s the exploration of Africa had begun in earnest, with finding the source of the Nile considered a primary objective. For some, the biblical connotations were crucial. Livingstone wondered whether it might be possible to navigate the Nile from the Mediterranean to Central Africa. Other explorers such as Speke, Burton, Baker and Grant were arguing over the source. Livingstone believed he could find it, and so restore his reputation. With this hope in mind, in 1866 he set off on the third of his expeditions. This time he took no European companions, but wandered around the lands to the west and south of Lake Tanganyika with African employees as companions. He was often sick and frequently required help from Arabic slavers. It was in 1871 at Ujiji, on the shores of Lake Tanganyika, that the journalist Henry Morton Stanley ‘found’ the doctor. Livingstone refused to return to Britain and continued his explorations in spite of increasing sickness and deteriorating mental state. Oliver Ransford's biography ‘David Livingstone: The Dark Interior’ (Ransford, 1978) interprets Livingstone's life through a diagnosis of his being cyclothymic, suffering a mild form of manic depression. He saw how Africans would have seen him during his last months,

‘[*They*] *became familiar with the haggard, bearded, benign, ageing man who was often hungry and sick, and yet for some incomprehensible reason wandered from one village to another, halting only to rest and ask innumerable questions or speak of a mysterious redeemer who was his master*’.

In May 1873, Livingstone died. It seems he literally bled to death. Although the exact cause is not known, it is possible that a combination of amoebic dysentery, bilharzia and the haemorrhoids that had afflicted him for many years ran their course. For his last days he was ‘*reduced to a skeleton*’, according to his notebook. Horrace Waller collated his notebooks into two volumes outlining these ‘Last Journeys’ (Livingstone, 1874). His small band of African employees carried him to Chief Chitambo's village at Ilala, in the swamp lands near Lake Bangweulu, in today's Northern Zambia. There he died. His companions buried his heart under a Mpundu tree, part of which now resides in the Hunterian Museum's collection at the University of Glasgow. They then embalmed his body and carried it across Africa to Bagamoyo, in modern Tanzania. From here it was returned to Britain, and eventually identified by the arm broken in the lion attack some 30 years before, and interred in Westminster Abbey in 1874. His burial stone includes a variation on a quote taken from his late notes, dramatically emphasizing his call to arms against the slave trade,

‘*All I can add in my solitude, is, may heaven's rich blessing come down on every one, American, English or Turk, who will help heal this open sore of the world*’.

## LIVINGSTONE AND PARASITIC DISEASE

Inevitably, spending 30 years in Africa and travelling across 30 000 miles of rough terrain, Livingstone was exposed to a multitude of parasites. Moreover, as a doctor, it was his business to understand and intervene against sickness. His writings are replete with instances where he describes his own illness as well as those of others. He recorded cases we can now diagnose as elephantiasis, hookworm, leprosy, yellow fever and many others. His observation that relapsing fever was transmitted by the tampan tick (now known to be caused by *Borrelia* bacteria) helped to show the way for understanding the key role of arthropods in transmission of disease. For malaria too he noted, explicitly, that the presence of mosquitoes correlated with the disease, although he continued to subscribe to the commonly held view that breathing of putrid air from swamps was the cause.

An intrepid generation of Scottish physician-scientists inspired by Livingstone went on to systematically identify the causes of many of these tropical diseases and the arthropod vectors that transmitted them (Cox, 1996, 2002 and this issue; Barrett *et al.* 2006).

## LIVINGSTONE AND MALARIA

Malaria, in the 19th century, as now, was the main parasitic disease responsible for morbidity and mortality in countries where the parasite is endemic. Today, rigorous intervention activities have seen a dramatic fall in incidence, although numbers afflicted still run beyond 200 million and many hundreds of thousands, mainly children, die from malaria each year (Marsh, 2016). Long before Livingstone, it was clear that ‘African fever’ was the primary obstacle confronting European explorers. As a doctor, and someone trained in the scientific method, Livingstone learned to manage the threat offered by this disease.

Quinine, a product of the bark of the Peruvian cinchona tree, above all else allowed him to survive in Africa. Quinine had been used by Europeans since the sixteenth Century, being exported by Spanish colonists. Already by the 1820s Pierre Joseph Pelletier and Joseph Bienaime Caventou had isolated the active ingredient of the bark.

**Fig. 2.**
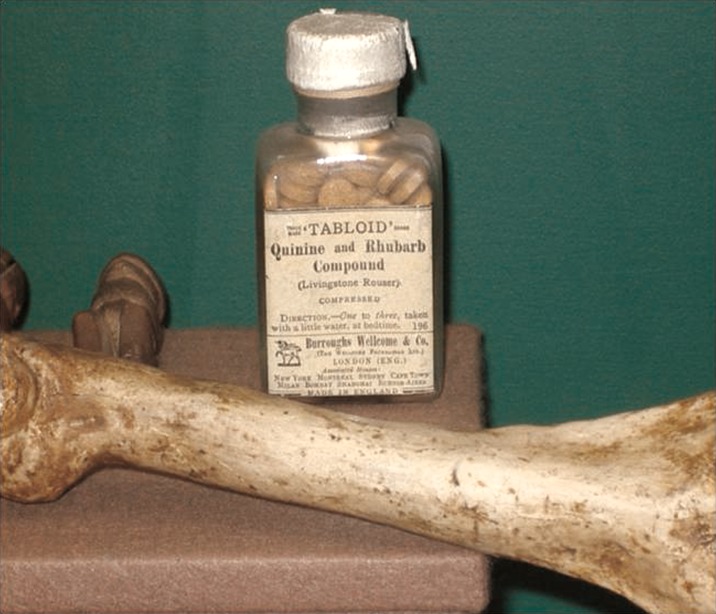
Livingstone's rousers. The quinine, rhubarb and jalap mixture that Livingstone concocted was later marketed by the Pharmaceutical Company Burroughs Wellcome & Co. This bottle is on display at the David Livingstone Centre in Blantyre. A cast of the bone broken in a lion attack is in the foreground.

Reading William Baikie's ‘Journals of the Niger Exploration’ (Baikie, 1856), where he describes the use of the drug, Livingstone was persuaded that quinine, taken with sherry, had been instrumental in preventing malaria for his predecessor. Therefore he purchased, from the Apothecaries Hall in London, huge quantities of the drug and took them with him to Africa. By trial and error he found it necessary to take doses until it elicited a ringing in the ears, a state known as cinchonism. Struck by reports of J.O. McWilliam (McWilliam, 1843) describing that tar-like deposits lined the gall bladder and blood vessels of malaria patients, Livingstone reasoned that an accumulation of this malaria-associated material could contribute to the disease. Regular movements of the bowels, he believed, would act to clear this material; hence, he developed a quinine-based formulation that also included rhubarb, jalap, from the root of the *Ipomoea* plant, and calomel, for added laxative effect to combat the disease. Burroughs Wellcome & Co. later marketed the formulation as ‘Livingstone's rousers’ (Fig. 2).

The availability, or not, of quinine was critical to Livingstone's survival and that of his companions and others arriving in Africa. For example, when he arrived at Ujiji in 1870, he found his quinine supplies gone and was awaiting death when Stanley, bringing fresh supplies, rescued him. The first missionaries to follow Livingstone to his mission site at Linyanti, the Reverend Holloway Helmore and Roger Price with their wives and children, were tragically struck by malaria. Ignorant of the health problems specific to sub-Saharan Africa, they had not taken their own quinine supplies and were unaware that Livingstone had left a stock of the drug at the mission. Only Price and two of the Helmore children survived. Later, other missionaries lead by Bishop Mackenzie, from the Universities’ Mission to Central Africa, arrived and established missions in the Shire Highlands, in today's Southern Malawi. Each was picked off with grisly inevitability by malaria, usually when isolated from supplies of the drug. Mary Livingstone too was killed by the disease, unable to keep quinine down due to her sickness.

Livingstone himself was regularly struck with malaria, recording his first bout as early as May 1853. He noted 27 bouts during his trek from Central Africa to Angola and continued to suffer while coming back. He made detailed records of the symptoms, the fever, its periodicity and responses to quinine. It was in 1880, 7 years after Livingstone's death, that French doctor Alphonse Laveran identified the malaria parasites in infected blood. In 1897, influenced by Livingstone's distant relative, Sir Patrick Manson (Manson-Bahr and Alcock, 1927), Sir Ronald Ross (Nye and Gibson, 1997), while working in India, finally proved the mosquito link. Ross found the parasites after a heroic effort, studying thousands of different mosquitoes before finally seeing pigment-bearing parasites in the dapple-winged *Anopheles* type. Manson had himself shown how *Aedes* mosquitoes transmit the filaria worms that cause lymphatic filariasis in 1877, and is widely recognized as ‘the Father of Tropical Medicine’, having gone on to found the London School of Tropical Medicine (now London School of Hygiene and Tropical Medicine) in 1899. Ross was the first Briton to win a Nobel Prize in 1902 and was a founding lecturer at the Liverpool School of Tropical Medicine that opened a year before the London School.

During the Zambezi expedition, John Kirk kept detailed notes on the incidence and symptoms of malaria suffered by the expedition members. Dr Charles Meller took on that role in February 1861 and published detailed accounts of his and Kirk's observations of fever during the expedition (Meller, 1862, 1864), including the occurrence of blackwater fever (where malaria pigment leaches into urine), treatment regimens and the relative propensity of Europeans and locals to suffer from the disease. Europeans were more vulnerable: an early indication of acquired, but not sterilizing, immunity to the disease.

It is a great pity that Kirk never published his own account of the Zambesi expedition. His discoveries were manifold and are available in his posthumously published notebooks (Foskett, 1965). In addition to his prowess as a doctor and naturalist, Kirk's political skills allowed him, as British consul in Zanzibar, to negotiate the deal with the Sultan there that effectively ended the slave trade in East Africa in 1873, the year Livingstone died. Kirk's daughter Helen married Major-General Henry Brooke Hagstromer Wright, brother of the great scientist Sir Almroth Wright, mentioned in this special edition as the mentor of Sir William Leishman (see article in this special issue by Heather Vincent). Although not the first to use quinine, Livingstone's fame and the reverence in which he was held helped assure systematic use of the drug as Britain extended her colonial rule in the tropics. The drug, still used today, also inspired the production of other drugs, such as chloroquine, a cheap, chemically made quinoline-based alternative. The emergence of resistance to chloroquine has rendered it useless across many parts of the world, but other drugs are available. Many of these are based on a Chinese herbal remedy, artemisinin, of which Livingstone would have approved, given his frequent and diligent experimentation with possible herbal cures for malaria and other diseases. New medicines are also appearing at an impressive rate in efforts led by the Medicines for Malaria Venture (MMV) (Wells *et al.* 2015), as modern pharmaceutical chemistry is applied to the disease.

## THE TAMPAN TICK AND RELAPSING FEVER

The influence of ‘Missionary Travels’ (Livingstone, 2012) in Britain cannot be overestimated. Missionaries left Britain for Africa in droves, and others were influenced too. The degree to which Livingstone's observations, outlined below, influenced Patrick Manson in showing how the brown mosquito of Amoy, *Culex fatigans* (now *Culex quinquefasciatus*) transmits lymphatic filariasis, and, in turn, Ross's discovery of *Anopheles* in transmission of malaria, cannot be certain. However, Livingstone made a compelling case on the transmission of disease by tsetse flies (see below), and also on human relapsing fever that followed a bite by a tampan tick. This offered a widespread audience their first glimpse of how arthropods can transmit disease. Livingstone wrote (Livingstone, 1857),

‘*When sleeping in the house of the commandant, an insect, well known in the southern country by the name Tampan, bit my foot. It is a kind of tick, and chooses by preference the parts between the fingers or toes for inflicting its bite. It is seen from the size of a pin's head to that of a pea, and is common in all the native huts in this country. It sucks the blood until quite full, and is then of a dark blue color, and its skin so tough and yielding that it is impossible to burst it by any amount of squeezing with the fingers. …..I shall detail the effects of the bite. These are a tingling sensation of mingled pain and itching, which commences ascending the limb until the poison imbibed reaches the abdomen, where it soon causes violent vomiting and purging. Where these effects do not follow, as we found afterward at Tete, fever sets in; and I was assured by intelligent Portuguese there that death has sometimes been the result of this fever. The anxiety my friends at Tete manifested to keep my men out of the reach of the tampans of the village made it evident that they had seen cause to dread this insignificant insect. The only inconvenience I afterward suffered from this bite was the continuance of the tingling sensation in the point bitten for about a week*’.

The great Glaswegian pathologist William Leishman, after whom *Leishmania* parasites are named (see paper in this special issue by Heather Vincent), in 1917 made a detailed study of the *Borrelia* species that are transmitted by the tick and made great inroads into understanding relapsing fever, which is still widespread, but relatively neglected, today. A related borrelial infection, Lyme disease, also transmitted by ticks, is in contrast the subject of increasing public concern in many parts of the world where its incidence is on the rise (Cutler, 2015).

## THE TSETSE FLY AND THE AFRICAN TRYPANOSOMIASES

Human African trypanosomiasis, or sleeping sickness, is a disease confined to sub-Saharan Africa that was once referred to as the ‘Colonial Disease’ (Lyons, 2002). It flared up in dramatic epidemics following the European colonization of the continent, as Africans were coerced to inhabit tsetse infested lands they had otherwise avoided through generations of experience. It is estimated that one third of the population of Uganda was felled by the disease in the early 20th century (Headrick, 2014). The disease is caused by trypanosomes that are transmitted by tsetse flies. Two forms of the disease are recognized, one in the East and Southern Africa caused by *Trypanosoma brucei rhodesiense* and the other, endemic in Central and West Africa caused by *T. b. gambiense*. Livingstone did not report any obvious case of human trypanosomiasis and, generally speaking, there is little to suggest the disease was found in East and Southern Africa at the time. One theory has proposed that it was infected people from Central Africa who had accompanied Henry Morton Stanley on his expedition from West to East Africa in 1886–1888 that carried the disease there (Headrick, 2014). However, this is improbable, since it was the gambiense form of the disease that was endemic to the Congo region (and still is), while evidence indicates that the Ugandan epidemic was caused by *T. b. rhodesiense*. The latter might instead have newly evolved as a distinct sub-species of *T. b. brucei* (an animal infective species) through the emergence of the so-called serum resistance-associated gene, which allows these parasites to avoid a lytic factor in human blood that normally kills *T. b. brucei* and other trypanosome species that do not have mechanisms to bypass its lethal effects.

**Fig. 3.**
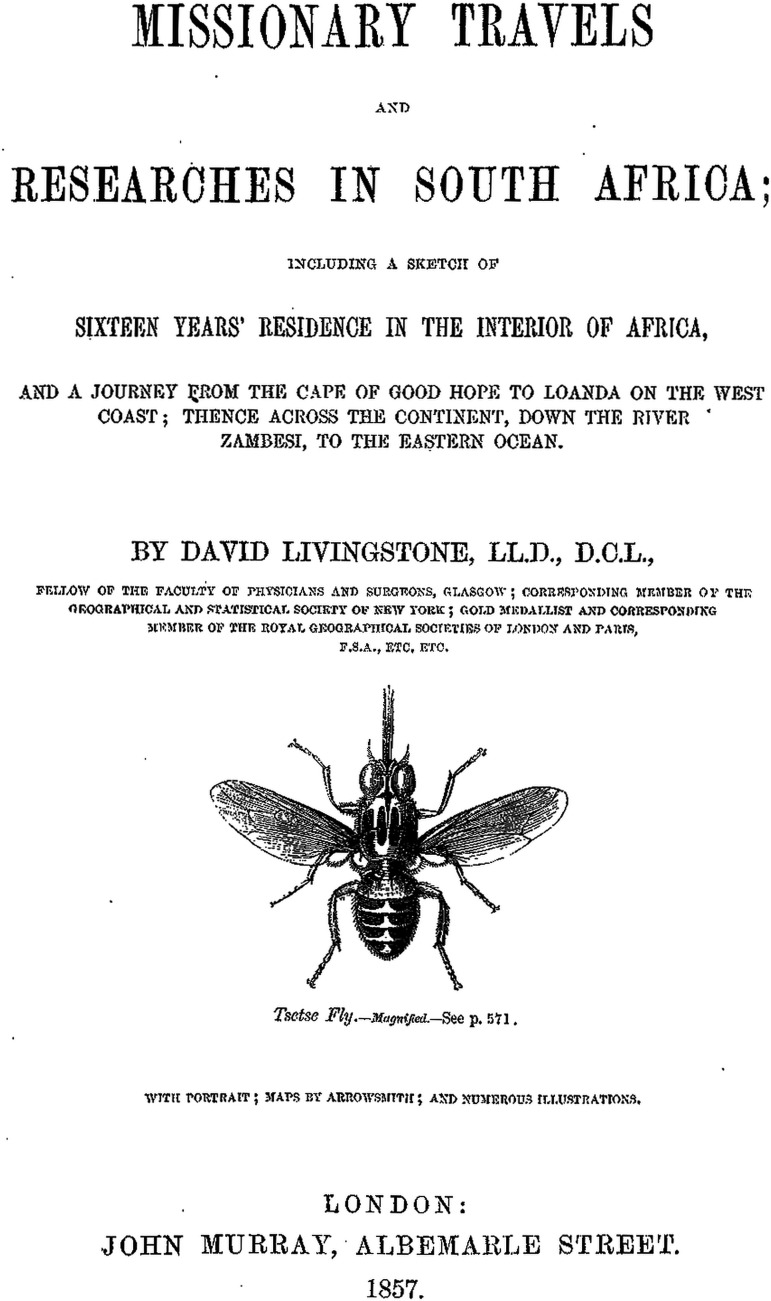
The frontispiece of ‘Missionary Travels and Researches in South Africa’ by Livingstone.

Livingstone was acutely aware that tsetse flies (*Glossina* spp.) transmitted a disease. African populations had co-evolved with the tsetse fly and, as the Bantu tribes migrated across Africa as cattle herders, their routes depended upon tsetse free corridors (Mackenzie, 1990) and settlement of cattle-herding populations was influenced by the fly's presence. Roualeyn Gordon-Cumming, who preceded Livingstone trekking north from the Cape, noted losses of horses and oxen in tsetse-infested regions (Mackenzie, 1990). Livingstone actually treated one of his horses following a tsetse bite (see later) and Frank Vardon, who visited Livingstone in Africa in 1846, brought a tsetse fly specimen back to London. The frontispiece of Livingstone's ‘Missionary Travels’ shows a tsetse fly (Fig. 3), testimony to the importance he placed on these insects in hindering economic development, in general, and European colonization, in particular, in Africa. He noted how *Glossina morsitans* resembled a common house-fly in size, and depicted its anatomy and behaviour in some detail.

In the areas he visited, however, Livingstone observed how humans, as well as wild animals, seemed to be immune to the ‘fly disease’, as he called it, and it was exclusively domesticated species that succumbed. The disease is today also known as nagana and is caused by the three non-human infective species *Trypanosoma congolense, Trypanosoma vivax* and *T. b. brucei*. Referring to the tsetse fly Livingstone wrote (Livingstone, 1857),

‘*Its peculiar buzz when once heard can never be forgotten by the traveler whose means of locomotion are domestic animals; for it is well known that the bite of this poisonous insect is certain death to the ox, horse, and dog*’.

In his first major exploratory trip to Lake Ngami, Livingstone lost forty-three oxen to tsetse flies. In ‘Missionary Travels’ he goes on,

‘*The poison does not seem to be injected by a sting, or by ova placed beneath the skin; for, when one is allowed to feed freely on the hand, it is seen to insert the middle prong of three portions, into which the proboscis divides, somewhat deeply into the true skin; it then draws it out a little way, and it assumes a crimson color as the mandibles come into brisk operation. The previously shrunken belly swells out, and, if left undisturbed, the fly quietly departs when it is full. A slight itching irritation follows, but not more than in the bite of a mosquito. In the ox this same bite produces no more immediate effects than in man. It does not startle him as the gad-fly does; but a few days afterward the following symptoms supervene: the eye and nose begin to run, the coat stares as if the animal were cold, a swelling appears under the jaw, and sometimes at the navel; and, though the animal continues to graze, emaciation commences, accompanied with a peculiar flaccidity of the muscles, and this proceeds unchecked until, perhaps months afterward, purging comes on, and the animal, no longer able to graze, perishes in a state of extreme exhaustion. Those which are in good condition often perish soon after the bite is inflicted with staggering and blindness, as if the brain were affected by it. Sudden changes of temperature produced by falls of rain seem to hasten the progress of the complaint; but, in general, the emaciation goes on uninterruptedly for months, and, do what we will, the poor animals perish miserably*’.

He continues to describe observations at post-mortem in forensic details and concludes,

‘*These symptoms seem to indicate what is probably the cause, a poison in the blood, the germ of which enters when the proboscis is inserted to draw blood. The poison-germ, contained in a bulb at the root of the proboscis, seems capable, although very minute in quantity, of reproducing itself, for the blood after death by tsetse is very small in quantity, and scarcely stains the hands in dissection*’.

In anticipation of our understanding that the trypanosome can survive inside the vertebrate host through its remarkable process of antigenic variation (Horn, 2014), where sequential expression, one at a time, of up to a thousand different coat proteins that cover the parasites, allows them to avoid obliteration by the immune system, he noted,

‘*Inoculation does not insure immunity, as animals which have been slightly bitten in one year may perish by a greater number of bites in the next*’.

We now known that each of those coat proteins elicits a separate antibody response – so each new variant loses susceptibility to antibodies targeted to the previous variant.

As mentioned before, most of Livingstone's travelling was carried out on foot and horses or oxen were used to pull carts. However, these animals did not survive long because of the tsetse bites. African buffalo (*Syncerus caffer*), instead, Livingstone noted, were tolerant of the tsetse bite, a phenomenon now known as trypanotolerance. It has been suggested that xanthine oxidase in buffalo blood is the factor responsible for this animal's ability to resist the trypanosomes transmitted by tsetse flies (Muranjan *et al.* 1997). Unfortunately, the African buffalo was refractory to domestication. Indian buffalo (*Bubalus bubalis*), however, were routinely used as draught animals, being less aggressive and amenable to exploitation. Consistent with his scientific thinking, Livingstone brought several Indian buffalo with him to Africa for the final expedition, only to find that trypanotolerance that had evolved in African buffalo was not manifest in the Indian type. Today we understand this to be a result of the co-evolution of the African buffalo (and of other indigenous breeds as well as wild African fauna) with trypanosomes, whilst the Indian buffalo was under no such evolutionary pressure. The imported buffalo soon succumbed to the tsetse bite.

While nagana continues to exert a profound effect on agricultural production in sub-Saharan Africa, the incidence of sleeping sickness has today fallen to just a few thousand cases. Central to this success in bringing the human disease under control has been the effective use of drugs. The therapy of trypanosomiasis also has roots stemming back to Livingstone. In 1858, he wrote a letter to the British Medical Journal (Livingstone, 1858) describing how the use of arsenic oxide (Fowler's solution) to treat a horse afflicted with fly disease had failed, responding to a letter proposing its use. In a prose account with a style that would help greatly in reading scientific reports today he said,

‘… *A mare belonging to Mr. Gordon Cumming was brought to Kolobeng, after prolonged exposure to the bite of the insect; and, as it was unable to proceed on the journey southward, its owner left it to die. I gave it two grains of arsenic in a little barley daily for about a week, when an eruption resembling small-pox appeared. This induced me to discontinue the medicine; and, when the eruption disappeared, the animal's coat became so smooth and glossy that I imagined I had cured the complaint; for, after the bite is inflicted, the coat stares as if the animal were cold. … About two months after this apparent cure, the coat began to stare again; but this time it had remarkable dryness and harshness. I tried the arsenic again; but the mare became like a skeleton, and refused to touch the barley. When I tried to coax her, she turned her mild eye so imploringly, and so evidently meaning, “My dear fellow, I would rather die of the disease than of the doctor”, that I could not force her. I got her lifted every morning to feed, and saw her at last perish through sheer exhaustion and this was nearly six months after the bite was inflicted*’.

It was some 40 years after Livingstone had written about fly disease that David Bruce demonstrated beyond doubt that the causative agent of human African trypanosomiasis in Uganda was the trypanosome (similar to the organisms he had previously shown to be responsible for nagana in South Africa).

The Italian Alberto Castellani had actually found trypanosomes in Ugandan sleeping sickness patients prior to Bruce, but having favoured a streptococcal cause, Bruce took the credit, much to Castellani's chagrin. Castellani's place in history was dealt a blow when he became personal physician to Italian dictator Benito Mussolini (Keynes, 1994) during *Il Duce's* visits to Britain before the Second World War. After the war Castellani was stripped of the knighthood bestowed upon him.

In 1905, influenced by Livingstone's experiments with arsenic (Fowler's solution) in trying to treat the horse reported above, Harold Wolferstan Thomas and Anton Breinl showed the trypanocidal activity of the organic arsenical atoxyl (Riethmiller, 2005). Paul Ehrlich then developed arsenical-based compounds further and created salvarsan. The drug found infamy when used for syphilis, primarily due to side-effects related to its oxidized products. Today, the arsenical melarsoprol is still the only drug available to treat stage 2 rhodesiense sleeping sickness (the form prevalent in East and Southern Africa, which is referred to as stage 2 once parasites have established an infection in the brain). Its relative, cymelarsan, is also used to treat some forms of animal trypanosomiasis. It is extraordinary that 158 years after Livingstone had discussed uses of arsenic to treat trypanosomiasis we are still using it! Fortunately, modern efforts to develop new drugs led by the Drugs for Neglected Diseases initiative (DNDi) offer great promise in bringing forward new orally available drugs to treat stage 2 human African trypanosomiasis (Bilbe, 2015).

## LIVINGSTONE AND PARASITIC HELMINTHS

Livingstone became familiar with helminth infections of man and animals. Although his ability to find infections in humans was hampered, as autopsy in people was unacceptable according to African custom at the time, he had the possibility to dissect animals. This allowed him to identify many macroparasitic helminths. He also noted the strange habit of some people to eat earth, a condition known as geophagy. These individuals, he remarked, were anaemic, manifesting through a loss of red colouration in fingernails. Patients were emaciated with swollen feet. Among locals the condition was known as ‘Safura’ and was clearly due to hookworm infection. Livingstone could identify only the symptoms, microscopic examination of feces not then being in routine diagnostic use. Hookworms live in the small intestine and in sub-Saharan Africa the species *Necator americanus* is endemic. The worms suck blood from the host, impairing their nutritional status. Loss of iron is a principal result of infection. The disease was endemic in the southern USA into the 20th century. It was widely considered to be responsible for a profound indolence that afflicted many inhabitants, black and white, in the southern states. The campaign launched by J. D. Rockefeller in 1910 through the ‘Rockefeller Sanitary Commission for the Eradication of Hookworm Disease’ was among the first great public health initiatives and was a profound success (Elman *et al.* 2014).

Today, over half a billion people still carry hookworms globally. It is one of the group of organisms known as the soil transmitted helminths (STHs) that also includes *Ascaris lumbricoides* and *Trichinella spiralis*. Wearing shoes greatly reduces the risk of becoming infected, which occurs when worm larvae burrow through the skin.

Livingstone also noted whipworm parasites (*Trichuris trichiura*), which are spread when people eat food contaminated with worm eggs. Eggs hatch and develop to adults in the large intestine, reaching up to 4 cm in length as adults. When large worm burdens accumulate, stomach aches and diarrhoea and lethargy can ensue. Microscopic identification of eggs in feces is the usual way to diagnose the disease today, and drugs such as albendazole and ivermectin are highly effective. However, there are still over 600 million people affected today (Hawdon, 2014). Although when there are few worms the affliction may be asymptomatic, very heavy worm burdens can cause the rectum to prolapse, occasionally teaming with worms, an affliction noted by Livingstone (Livingstone, 1857) and called ‘Shirugosa’ among locals.

One of his team, Livingstone noted, had ‘*an insect in the eye*’. Michael Gelfand, whose 1957 biography ‘Livingstone the Doctor’ offers an excellent summary of Livingstone's medical life (Gelfand, 1957), surmised this was the filarial worm *Loa loa*, whose larvae and adults reside in subcutaneous regions beneath the skin. They are transmitted by *Chrysops* horseflies when they bite humans. *L. loa* parasites travel from the bite site through subcutaneous tissues where they cause inflammation of the skin; if they remain in one place they cause a lesion commonly referred to as ‘Calabar swelling’. They can also migrate through the body and infect the eye, causing swelling. It is not clear that what Livingstone saw would really have been *L. loa* though, as this parasite is more prevalent in West Africa.

While dissecting animals, Livingstone was struck by the parasitic burden. For example, he found a bundle of red worms in the oesophagus of a butchered zebra, almost certainly cyathostomes, the common redworm that is a major equine parasite. The zebra's intestine too was teaming with nematodes. Later, he noted flukes in the liver of a dead cow. The following is another section from ‘Missionary Travels’,

‘*Inquiries among the Bushmen and Bakalahari, who are intimately acquainted with the habits of the game, lead to the belief that many diseases prevail among wild animals. I have seen the kokong or gnu, kama or hartebeest, the tsessebe, kukama, and the giraffe, so mangy as to be uneatable even by the natives*’.

‘*I once found a buffalo blind from ophthalmia standing by the fountain Otse; when he attempted to run he lifted up his feet in the manner peculiar to blind animals. The rhinoceros has often worms on the conjunction of his eyes; but these are not the cause of the dimness of vision which will make him charge past a man who has wounded him, if he stands perfectly still, in the belief that his enemy is a tree. It probably arises from the horn being in the line of vision…*’.

‘*All the wild animals are subject to intestinal worms besides. I have observed bunches of a tape-like thread and short worms of enlarged sizes in the rhinoceros. The zebra and elephants are seldom without them, and a thread-worm may often be seen under the peritoneum of these animals. Short red larvae, which convey a stinging sensation to the hand, are seen clustering round the orifice of the windpipe (trachea) of this animal at the back of the throat; others are seen in the frontal sinus of antelopes; and curious flat, leech-like worms, with black eyes, are found in the stomachs of leaches*’.

## LIVINGSTONE AND SCHISTOSOMIASIS

Of all the helminth infections, bilharzia, or schistosomiasis, deserves particular attention with regard to Livingstone, since chronic schistosomiasis may have played a key role in his death. It was the morning of 1st May 1873 that Majiwa, one of the Africans travelling with him in his final days, found Livingstone kneeling beside his bed. He was dead, possibly in prayer, but given the excruciating pains in his intestines about which he had been complaining in the preceding days, maybe in an effort to relieve those symptoms. He had written in his journal just a few days before,

‘*I am pale, bloodless and weak from bleeding profusely ever since 31^st^ March last. An artery gives off a permanent stream and takes away my strength*’.

He had suffered from haemorrhoids for many years, declining the opportunity to have them removed because of the embarrassment he feared would follow. Dysentery, possibly amoebic, was a constant problem, and it is difficult to imagine he was free of intestinal helminths. Drinking freely when necessary, chances to become infected were common. Crossing the Kalahari Desert in the 1840s, he remarked,

‘*I have drunk water swarming with insects, thick with mud and putrid with rhinoceros urine and buffaloes’ dung, and no stinted drafts of either …*’

Schistosome infection seems inevitable. Tramping through the swamps around Lake Bangwuelu and swimming in lakes would have exposed him repeatedly to the cercariae larvae, endemic throughout the Great Lakes region. Lake Nyasa was, he described, ‘*a heavenly place to bathe*’. The freshwater snails that act as vectors of the disease are widespread in this part of Africa. Although today an effective treatment, praziquantel, can kill adult worms, there was no treatment in Livingstone's time. The German physician, Theodore Bilharz (hence the common name of bilharzia given to the disease), had described the causative worms in Egypt in 1851 (Hagan, 2009), but regular diagnosis and treatment were not available during Livingstone's expeditions.

The cercariae larvae enter the bloodstream and transform, via schistosomulae, into adult worms. Females and males form reproductive couples and the females produce hundreds of eggs each day. Some of these escape through urine or feces into water and hatch into miracidia larvae that infect snails before converting to the cercariae that infect new hosts. Other eggs lodge in tissues and our immune response to them ends up damaging the organs in which the eggs are found. The more worms, the more eggs; the more eggs, the more damage. Livingstone wrote of his own haematuria, describing his urine as being of brick-dust colour during his first trans-Africa trek. His children and various of his travelling companions too suffered from blood in their urine, pointing to exposure. Continuous acquisition of worms throughout his 30 years in Africa would have produced a large worm burden in Livingstone. When his body was opened by his companions after his death, in order to embalm him for a long trek back across Central Africa, before shipping back to England, they found what they described a blood clot as large as a fist. More likely this was an enlarged spleen, a product of recurrent malaria and chronic schistosomiasis.

## LIVINGSTONE AND DARWIN: CONTEMPORARY NATURALISTS

Livingstone's extraordinary capabilities as a naturalist awakened the imaginations of many British people to the exciting diversity of wild life abroad. It is seldom considered that he was, in fact, a direct contemporary of Charles Darwin. We have not been able to find evidence the two met. Darwin stopped at Cape Town on his way back from the Beagle's voyage in 1836, well before Livingstone arrived in Africa. The two men, however, shared a publisher, John Murray, official publisher of the Royal Geographical Society, who published Livingstone's ‘Missionary Travels’ in 1857, 2 years before he finally got Darwin's ‘On the Origin of Species’ (Darwin, 1859) to print. Although Darwin was from a wealthy background, while Livingstone's upbringing was impoverished, both men had studied medicine in Scotland (Darwin in Edinburgh, Livingstone in Glasgow), before moving to England to pursue studies in theology (Darwin at Cambridge, Livingstone with the LMS). Both sailed to distant lands to explore natural history. Both corresponded with the great figures of the scientific establishment of the day, men like Richard Owen at the Natural History Museum, Joseph Hooker at Kew Gardens, and geologist Charles Lyell and Roderick Murchison at the Royal Geographical Society. Critically, both were also driven by a fundamental desire to combat slavery (Desmond and Moore, 2009). Given Livingstone's observations then, it may seem curious that he responded to Darwin's theory of evolution in a letter to Richard Owen by expressing an inability to ‘*see any struggle for survival on the wide continent (of Africa)*’. However, at this time Livingstone had not yet received a copy of Darwin's book nor had time to reflect on its implications.

It has been speculated that Darwin might have died from chronic Chagas’ disease caused by the American trypanosome, *Trypanosoma cruzi* (Adler, 1959). His remains, like Livingstone's, lie in Westminster Abbey. The advent of post-mortem genetic analysis has led to calls to exhume Darwin's body to seek signs of DNA from the causative trypanosome. Applying the same to Livingstone would likely be fruitless. His body was disembowelled, left to sun-dry and preserved with alcohol and salt before being wrapped in tar-impregnated cloth and carried a thousand miles to Bagamoyo, in modern day Tanzania. From here it was carried to Zanzibar and arrived back in England only a year later. The disfigured corpse was identified through the broken arm suffered in the lion attack many years earlier. Even if any DNA had survived the journey and subsequent burial, it is likely that the mixture of parasite species found within him would be so diverse that a positive link to a cause of death would be no more feasible than through piecing together causes related to the symptoms he so painstakingly provides in his notes. Irrespective of his cause of death, Dr Livingstone's pioneering work in tropical medicine directly influenced a generation of doctors, many of them fellow Scots, to seek the causes of tropical disease.
